# An Australian State-Based Cohort Study of Autoimmune Encephalitis Cases Detailing Clinical Presentation, Investigation Results, and Response to Therapy

**DOI:** 10.3389/fneur.2021.607773

**Published:** 2021-02-22

**Authors:** Andrew Swayne, Nicola Warren, Kerri Prain, David Gillis, Cullen O'Gorman, Benjamin K-T. Tsang, Claire Muller, Simon Broadley, Robert J. Adam, Pamela McCombe, Richard C. Wong, Stefan Blum

**Affiliations:** ^1^Mater Centre for Neurosciences, Mater Hospital, Brisbane, QLD, Australia; ^2^School of Medicine, The University of Queensland, Brisbane, QLD, Australia; ^3^Department of Neurology, Princess Alexandra Hospital, Brisbane, QLD, Australia; ^4^Department of Psychiatry, Princess Alexandra Hospital, Brisbane, QLD, Australia; ^5^Health Support Queensland, Pathology Queensland Central Laboratory, Division of Immunology, Royal Brisbane and Women's Hospital, Brisbane, QLD, Australia; ^6^Department of Neurology, Sunshine Coast University Hospital, Sunshine Coast, QLD, Australia; ^7^Department of Neurology, Royal Brisbane and Women's Hospital, Brisbane, QLD, Australia; ^8^Department of Neurology, Gold Coast University Hospital, Gold Coast, QLD, Australia; ^9^Centre for Clinical Research, The University of Queensland, Brisbane, QLD, Australia; ^10^Department of Immunology, Princess Alexandra Hospital, Brisbane, QLD, Australia

**Keywords:** autoimmune encephalitis, anti-NMDA-receptor antibody encephalitis, immune therapy, anti-glycine-receptor antibody associated disease, anti-CASPR2 encephalitis, anti-LGI1 encephalitis

## Abstract

**Introduction:** Autoimmune encephalitis is a disorder associated with antibodies directed against central nervous system proteins with variable clinical features. This study aims to add to knowledge of the disease by reporting the details of a cohort of patients with autoimmune encephalitis in Queensland, Australia.

**Methodology:** We surveyed patients with autoimmune encephalitis diagnosed and managed through public hospitals in Queensland, Australia between 2010 and the end of 2019. Cases were identified via case detection through a centralized diagnostic neuroimmunology laboratory (Division of Immunology, HSQ Pathology Queensland Central Laboratory, Brisbane, Queensland, Australia) and a survey of neurologists. Data including demographic details, clinical presentation, investigation results, treatments including immune therapy and outcomes was collected.

**Results:** Sixty cases of antibody positive autoimmune encephalitis were identified. Twenty-eight were of anti-NMDA-receptor encephalitis with other cases associated with antibodies against LGi1, Caspr2, glycine receptor, DPPX, GABA_B_ receptor, IgLON5, GFAP, and SOX1. The number of diagnosed cases, especially of anti-NMDA-receptor encephalitis has markedly increased over the period 2017 to 2019. Clinical presentations were marked by heterogeneous symptom complexes and prolonged hospital admissions. Imaging studies were largely normal or non-specific. There was a response to immune therapy and a low mortality rate. Most cases affected by this disorder were left with ongoing symptoms associated with mild disability.

**Conclusion:** Autoimmune encephalitis in Queensland, Australia is an increasingly common but complex clinical entity marked by heterogeneous presentations, response to immune therapy and outcome results marked by low mortality and incomplete recovery.

## Introduction

Recent years have seen autoimmune encephalitis become established as a potential diagnosis in patients presenting with a range of neurological and psychiatric symptoms. In 2007 Dalmau et al. detailed the clinical features of 12 women presenting with psychosis, seizure, memory disturbance, movement disorder, autonomic dysfunction and decreased level of consciousness, in association with ovarian teratoma and antibodies against N-methyl-D-Aspartate receptor (NMDA-R) (1).

Since then, understanding of autoimmune encephalitis has evolved and now represents a clinical spectrum of disease phenotypes, associated with more than 15 central nervous system protein targets (2). These antigenic targets are predominantly synaptic cleft channels or proteins and include leucine-rich glioma inactivated 1 (LGi1) (3, 4), contactin-associated-protein-like 2 (Caspr2) (5–7), glycine receptor (8, 9), gamma-aminobutyric acid-B receptor (GABA_B_R) (10, 11), dipeptidyl-peptidase-like protein 6 (DPPX) (12), immunoglobulin-like cell adhesion molecule 5 (IgLON5) (13–15), glial fibrillary acidic protein (GFAP) (16), and SRY-Box Transcription Factor 1 (SOX1) (17–19). The growing number of reported cases has broadened the clinical spectrum associated with autoimmune encephalitis (20).

Recent international cohort studies have analyzed single center autoimmune encephalitis experience with clinical presentations and management approaches (21), short-term prognosis in a Chinese center (22) and analyzed the high medical costs involved in autoimmune encephalitis presentations (23). There are emerging Australian autoimmune encephalitis data sets (24, 25). To date, these have focused on defining characteristics of autoimmune encephalitis in Australia with reference to specific investigation findings (26, 27), certain antibody subsets (24) or the relationship between autoimmune encephalitis and psychiatric presentations (25, 28).

In this study the authors look to build on recent developments in knowledge of autoimmune encephalitis by presenting findings from an Australian region-based analysis of cases. This incorporates autoimmune encephalitis presenting to tertiary neurology centers, psychiatric services and other hospitals within Queensland, Australia. This approach was enabled by the collation of cases through a central diagnostic neuroimmunology laboratory. There is specific focus within this dataset in assessing and comparing clinical data, therapeutic approach, treatment response and outcome measures across a range of autoimmune encephalitis subsets.

## Materials and Methods

Case detection was undertaken via ascertainment of antibody cases through a centralized diagnostic neuroimmunology laboratory (Division of Immunology, HSQ Pathology Queensland Central Laboratory, Brisbane, Queensland, Australia) and a survey of neurologists. Data was collected on identified cases via both their treating neurologist and through a retrospective chart audit.

Since 2010 to the present, all autoimmune encephalitis-related antibody and anti-neuronal antibody testing for Queensland public hospitals has been performed in a single laboratory (Division of Immunology, HSQ Pathology Queensland Central Laboratory). This is a diagnostic referral laboratory offering specialized neuroimmunology testing. The following assays were used for the detection of the various autoimmune encephalitis-related antibodies and anti-neuronal antibodies. Anti-NMDA-receptor IgG antibodies were detected in serum and cerebrospinal fluid (CSF) by indirect immunofluorescence using a commercial assay containing four biochips of primate hippocampus, primate cerebellum, fixed NR1-transfected human embryonic kidney 293 (HEK293) cells, and fixed non-transfected control HEK293 cells (IIFT: Glutamate Receptor Mosaic 3, Euroimmun, Lübeck, Germany). Antibodies against LGi1, Caspr2, GABA_B_R, a-amino-3-hydroxy-5-methyl-4-isoxazolepropionic acid receptor types 1 and 2 (AMPAR1 and AMPAR2, respectively), were detected in serum and CSF by indirect immunofluorescence using a commercial assay containing biochips of fixed HEK293 cells transfected with LGi1, CASPR2, GABA_B_R, AMPAR1, and AMPAR2 (IIFT: Autoimmune Encephalitis Mosaic 6, Euroimmun, Lübeck, Germany). Anti-glycine-receptor antibody analyses were performed by the Autoimmune Neurology Diagnostic Laboratory (Oxford, UK) using a cell-based assay of live HEK293 cells transfected to express homomeric alpha 1 glycine-receptor subunits (24).

Antibodies against SOX1, IgLON5, GFAP, and DPPX were screened in serum and CSF by indirect immunofluorescence using a commercial fixed composite slide containing primate cerebellum, primate cerebrum and murine stomach (NOVA Lite Monkey Cerebellum/Cerebrum & Mouse Stomach Slide Pack, Inova Diagnostics, San Diego, CA, USA). The presence of antibodies against SOX1 in serum and CSF was confirmed using a commercial immunoblot that contained SOX1 (EUROLINE Neuronal Antigens Profile PLUS RST (IgG), Euroimmun, Lübeck, Germany). The presence of antibodies of IgLON5 in serum and CSF was confirmed by indirect immunofluorescence using a commercial assay containing biochips of fixed HEK293 cells transfected with IgLON5 (IIFT: IgLON family member 5, Euroimmun, Lübeck, Germany). The presence of antibodies against GFAP in CSF was sent for confirmation by the Autoimmune Neurology Diagnostic Laboratory (Oxford, UK). Finally, the presence of antibodies against DPPX in serum and CSF was confirmed by immunoprecipitation studies, and this patient was previously reported in a series of patients with this antibody (12).

Case data was assessed by both a neurologist and neuropsychiatrist (AS and NW).

Adult patients (defined as >18 years of age at time of identification by the study authors) were included if there was a positive IgG NMDA-receptor antibody or other autoimmune encephalitis associated antibody in either serum or CSF (cerebrospinal fluid) and if their clinical features and investigations were consistent with autoimmune encephalitis. Seronegative cases were excluded. Individuals were excluded if younger than 18 years of age at the time of case identification, if the antibody detected had only been reported and/or confirmed by another diagnostic laboratory (with the exception of anti-Glycine-receptor antibodies, anti-DPPX and anti-GFAP), if the patient lived outside of Queensland at the time of case identification or if the autoimmune encephalitis was secondary to another neuroinflammatory or neuroinfectious condition such as anti-NMDA-receptor encephalitis complicating herpes simplex virus encephalitis. Cases with antibodies targeting intracellular or intracytoplasmic oncogenic antigens (ANNA-1/Hu, ANNA-2/Ri, PCA-1/Yo, PCA-2, anti-Ma/Ta, anti-amphiphysin, anti-GAD, and anti-CV2) were also excluded.

Ethical approval for this study was granted from Metro-South Human Research and Ethics Committee reference HREC/17/QPAH/423.

## Results

Sixty cases of autoimmune encephalitis meeting inclusion criteria were identified. Twenty-eight were of anti-NMDA-receptor encephalitis. The next most common subtypes were fourteen cases of anti-glycine-receptor related neurological disease and eight cases of anti-LGi1 encephalitis. There were three or fewer cases of autoimmune encephalitis with antibodies directed against Caspr2, DPPX, GABA_B_R, IgLON5, GFAP, and SOX1. Data related to the patients with anti-glycine-receptor related neurological disease has previously been presented (24).

**Figure 1:**
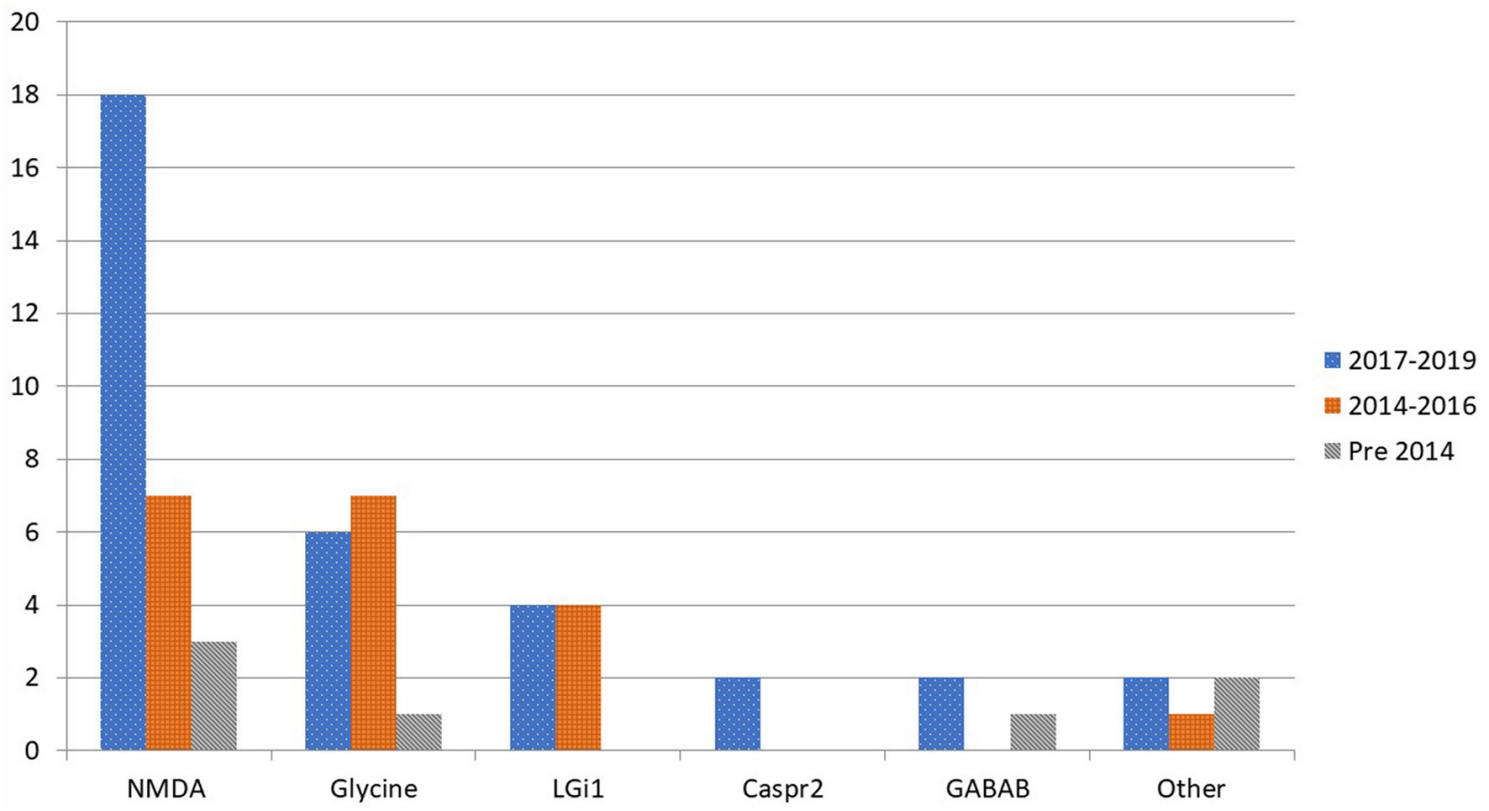
Distribution by time period of diagnosed cases in Queensland of autoimmune encephalitis by antibody subtype.

Figure 1 presents autoimmune cases diagnosed over time. Compared to the number of cases pre-2014 there is an increase of identified cases across all subtypes, most notably with the 18 cases of anti-NMDA-receptor encephalitis diagnosed during the 2017 to 2019 period.

Demographic data and past medical history of each autoimmune encephalitis subgroup is presented in Table 1. Across all subtypes there was a wide age at onset distribution (16–79 years old). Anti-NMDA-receptor encephalitis cases showed both a lower mean (35.25 years) and median (28 years) age of onset when compared to the other subtypes. There was a female to male predominance in the anti-NMDA-receptor encephalitis (55.5% female) and anti-glycine-receptor groups (78.6% female).

**Table 1:** Demographic and past medical history of autoimmune encephalitis cases by antigenic target.

	**NMDA-R**	**Glycine-R**	**LGi1**	**Caspr2**	**DPPX**	**GABA_**B**_R**	**IgLON5**	**GFAP**	**SOX1**
Number of cases	28	14	8	2	1	3	1	1	2
Age of onset range (years)	16–78	20–79	46–69	28–63	45–50	52–71	70–75	35–40	35–56
Mean age of onset (years)	35.25	44.1	58.25	59.5	N/A	63	N/A	N/A	45.5
Median age of onset (years)	28	42.7	57	59.5	N/A	66	N/A	N/A	45.5
Male: female ratio	10 to 18	3 to 11	5 to 3	1 to 1	N/A	1 to 2	N/A	N/A	2 to 0
Prior brain injury	4/28 (14.3%)	2/14 (14.3%)	1/8 (12.5%)	0/2 (0.0%)	0/1 (0.0%)	0/3 (0.0%)	0/1 (0.0%)	0/1 (0.0%)	0/2 (0.0%)
Pre-existing autoimmune condition	6/28 (21.4%)	1/14 (7.1%)	0/8 (0.0%)	0/2 (0.0%)	0/1 (0.0%)	0/3 (0.0%)	0/1 (0.0%)	0/1 (0.0%)	1/2 (50.0%)
Prior psychiatric history	10/28 (35.7%)	2/14 (14.3%)	2/8 (25.0%)	0/2 (0.0%)	0/1 (0.0%)	0/3 (0.0%)	0/1 (0.0%)	0/1 (0.0%)	1/2 (50.0%)
Ovarian teratoma	5/28 (17.9%)	1/14 (7.1%)	0/8 (0.0%)	0/2 (0.0%)	0/1 (0.0%)	0/3 (0.0%)	0/1 (0.0%)	0/1 (0.0%)	0/2 (0.0%)
Other pre-existing malignancy	0/28 (0.0%)	2/14 (14.3%)	0/8 (0.0%)	1/2 (50.0%)	0/1 (0.0%)	3/3 (100%)	0/1 (0.0%)	0/1 (0.0%)	0/2 (0.0%)

Prior brain injury was present in <15% of cases across the most represented subgroups. A pre-existing autoimmune condition was found in 21.4% of cases with anti-NMDA-receptor encephalitis. These conditions included Coeliac Disease, Autoimmune Spondyloarthritis, Type One Diabetes Mellitus, Vitiligo, Crohn's Disease, and Hashimoto's Thyroiditis. There was a higher percentage of past history of a psychiatric condition in the anti-NMDA-receptor encephalitis group than in the other groups (35.7%).

Ovarian teratoma was most common in the anti-NMDA-receptor antibody encephalitis and was found in 17.9% of cases. The three cases with anti- GABA_B_R encephalitis all had comorbid small cell lung cancer.

**Figure 2:**
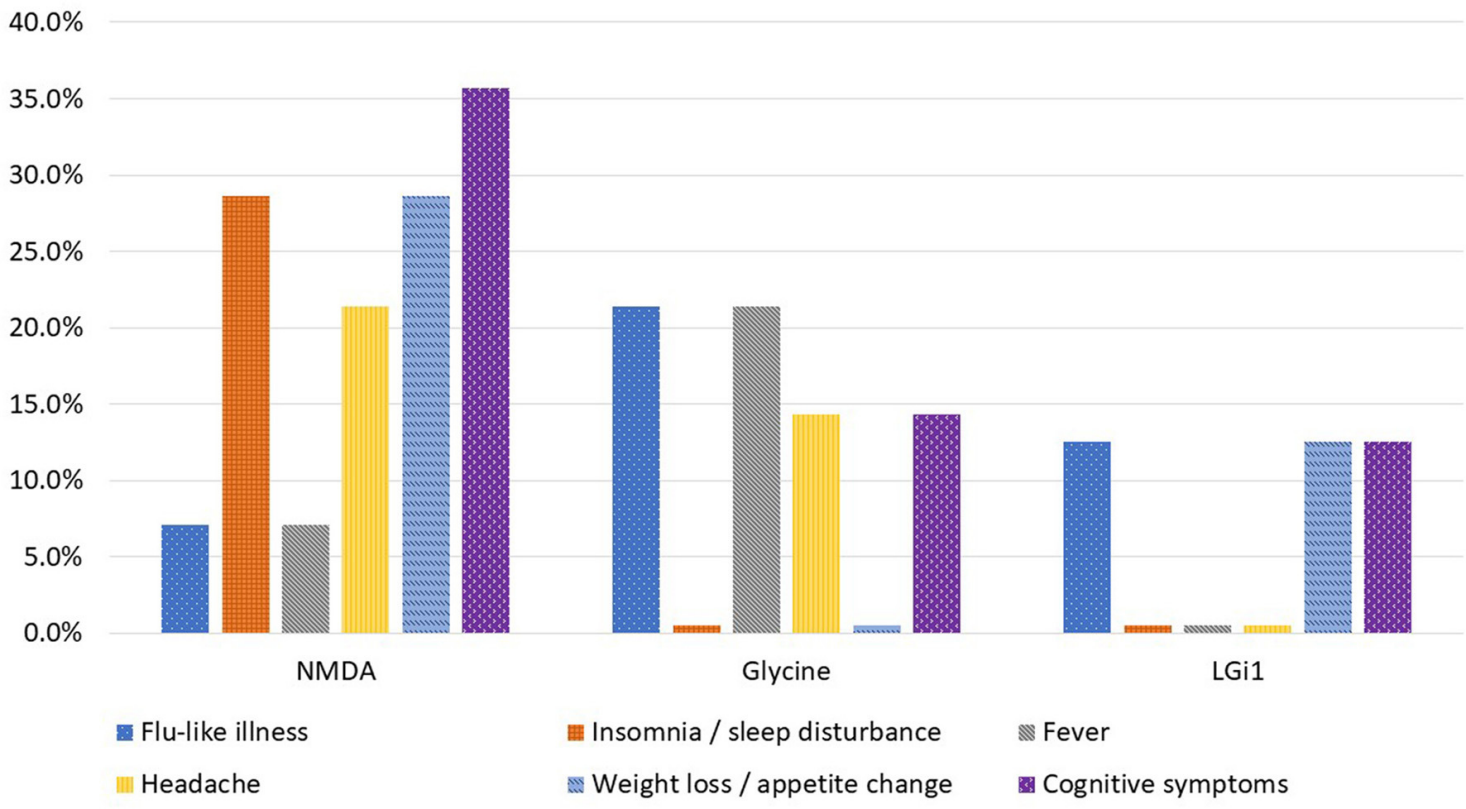
Percentage of cases who experienced prodromal symptoms by autoimmune encephalitis subtype.

Prodromal symptoms (flu-like illness, insomnia/sleep disturbance, fever, headache, weight loss/appetite change and cognitive symptoms) were present in all autoimmune encephalitis subtypes aside from anti-GABA_B_R encephalitis. Figure 2 illustrates the percentage of cases experiencing each prodromal symptom from the most common autoimmune encephalitis subgroups in this cohort study. Anti-NMDA-receptor encephalitis cases experienced cognitive change, sleep disturbance, headache and appetite change in 20% or more of cases. The glycine subgroup developed fever and flu-like symptoms in more than 20% of cases. In the anti-Caspr2 cases, 50% had at least one of the six prodromal symptoms. The anti-DDPX case only had prodromal appetite change. The anti-IgLON5 case developed a flu-like illness, dysphagia and sleep disturbance likely representing parasomnia. The anti-GFAP case had three prodromal symptoms (flu-like illness, sleep disturbance, and fever). In the anti-SOX1 encephalitis cases, 50% experienced flu-like illness and fever.

**Figure 3:**
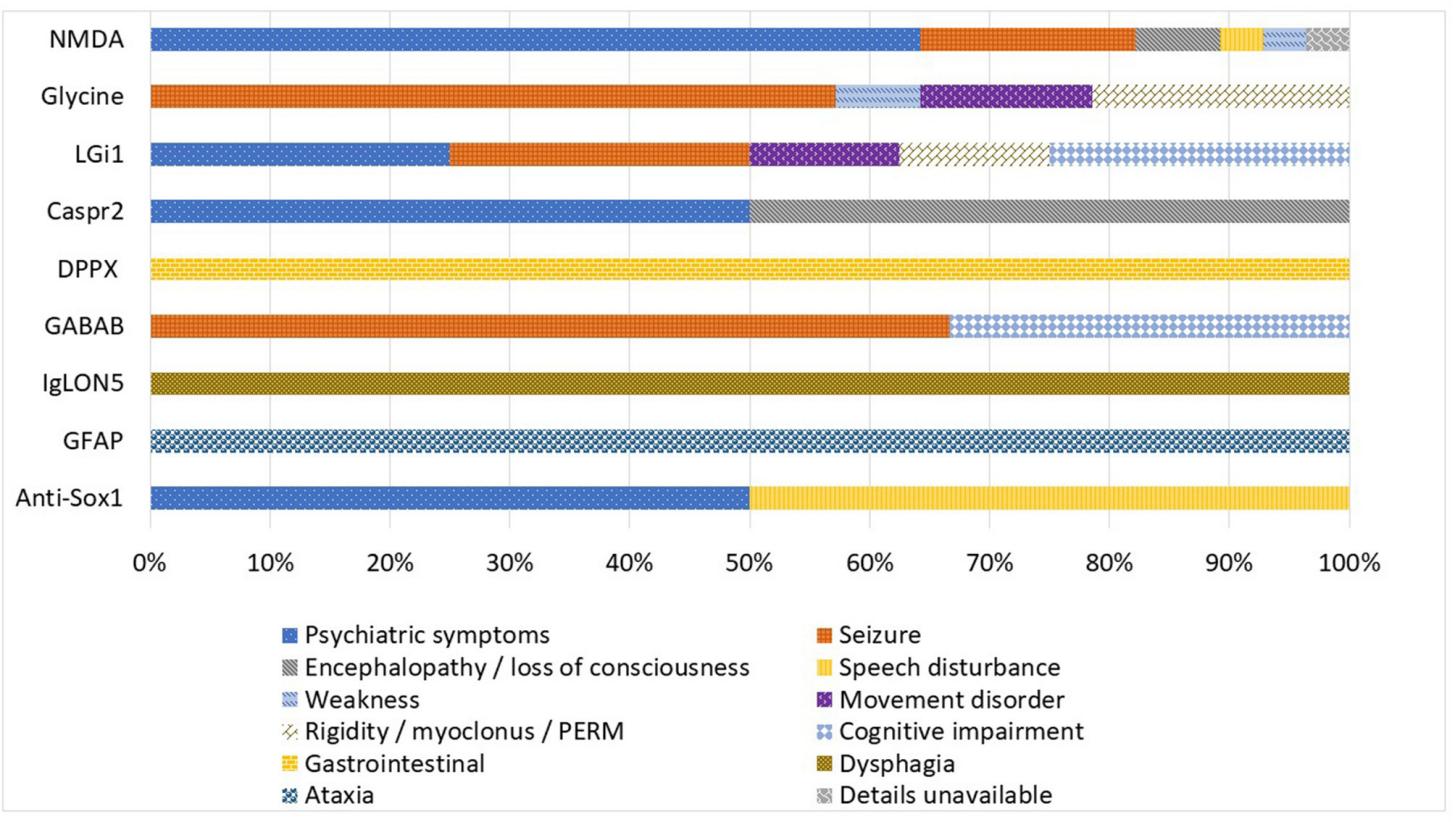
Autoimmune encephalitis subgroups by presenting symptom (expressed as percentage of cases).

There was a wide range of presenting symptoms across the Queensland autoimmune encephalitis cohort as illustrated in Figure 3. Psychiatric symptoms (64.3%) followed by seizure (17.9%) were the most common presenting symptom of anti-NMDA-receptor encephalitis cases. Anti-glycine-receptor associated disorder cases most commonly presented with seizure (57.1%) and Progressive Encephalomyelitis with Rigidity and Myoclonus (PERM) (21.4%). Psychiatric symptoms, seizure, cognitive symptoms and encephalopathy were common presentations across the anti-LGi1, anti-Caspr2, anti-GABA_B_R and anti-SOX1 subgroups. Unique presenting symptoms of gastrointestinal dysfunction, dysphagia and ataxia were seen in the cases with antibodies against DPPX, IgLON5, and GFAP, respectively.

Table 2 illustrates symptoms experienced by patients across their entire clinical course. In the anti-NMDA-receptor encephalitis group more than 35% of cases experienced cognitive impairment, mood disorder, auditory hallucinations, delusional thoughts and/or seizure. Within that same cohort there were additionally more than 15% of cases that displayed symptoms of visual hallucination, speech disturbance, weakness, autonomic dysfunction, and/or movement disorder.

**Table 2:** Symptoms present during disease course of autoimmune encephalitis cases by antigenic target.

	**NMDA-R**	**Glycine-R**	**LGi1**	**Caspr2**	**DPPX**	**GABA_**B**_R**	**IgLON5**	**GFAP**	**SOX1**
Cognitive impairment	25/28 (89.3%)	6/14 (42.9%)	5/8 (62.5%)	2/2 (100.0%)	1/1 (100.0%)	3/3 (100.0%)	0/1 (0.0%)	1/1 (100.0%)	2/2 (100.0%)
Mood disorder (elevated or depressed)	13/28 (46.4%)	2/14 (14.3%)	1/8 (12.5%)	1/2 (50.0%)	1/1 (100.0%)	1/3 (33.3%)	0/1 (0.0%)	0/1 (0.0%)	2/2 (100.0%)
Visual hallucination	9/28 (32.1%)	0/14 (0.0%)	0/8 (0.0%)	1/2 (50.0%)	1/1 (100.0%)	0/3 (0.0%)	0/1 (0.0%)	0/1 (0.0%)	0/2 (0.0%)
Auditory hallucination	11/28 (39.3%)	0/14 (0.0%)	1/8 (12.5%)	1/2 (50.0%)	0/1 (0.0%)	0/3 (0.0%)	0/1 (0.0%)	0/1 (0.0%)	1/2 (50.0%)
Delusional thoughts	15/28 (53.6%)	0/14 (0.0%)	1/8 (12.5%)	1/2 (50.0%)	1/1 (100.0%)	0/3 (0.0%)	0/1 (0.0%)	0/1 (0.0%)	1/2 (50.0%)
Seizure/s	17/28 (60.7%)	9/14 (64.3%)	6/8 (75.0%)	1/2 (50.0%)	1/1 (100.0%)	3/3 (100.0%)	0/1 (0.0%)	0/1 (0.0%)	1/2 (50.0%)
Status epilepticus	4/28 (14.3%)	4/14 (28.6%)	1/8 (12.5%)	0/2 (0.0%)	0/1 (0.0%)	0/3 (0.0%)	0/1 (0.0%)	0/1 (0.0%)	0/2 (0.0%)
Speech disturbance	5/28 (17.9%)	2/14 (14.3%)	0/8 (0.0%)	1/2 (50.0%)	0/1 (0.0%)	0/3 (0.0%)	1/1 (100.0%)	1/1 (100.0%)	0/2 (0.0%)
Weakness (including respiratory muscle weakness)	7/28 (25.0%)	1/14 (7.1%)	1/8 (12.5%)	1/2 (50.0%)	0/1 (0.0%)	1/3 (33.3%)	1/1 (100.0%)	1/1 (100.0%)	0/2 (0.0%)
Autonomic dysfunction	8/28 (28.6%)	1/14 (7.1%)	0/8 (0.0%)	2/2 (100.0%)	0/1 (0.0%)	1/3 (33.3%)	1/1 (100.0%)	1/1 (100.0%)	0/2 (0.0%)
Ataxia	4/28 (14.3%)	1/14 (7.1%)	0/8 (0.0%)	2/2 (100.0%)	0/1 (0.0%)	0/3 (0.0%)	0/1 (0.0%)	1/1 (100.0%)	0/2 (0.0%)
Movement disorder	7/28 (25.0%)	3/14 (21.4%)	1/8 (12.5%)	1/2 (50.0%)	0/1 (0.0%)	0/3 (0.0%)	0/1 (0.0%)	0/1 (0.0%)	0/2 (0.0%)
Altered sensation	2/28 (7.1%)	0/14 (0.0%)	0/8 (0.0%)	0/2 (0.0%)	0/1 (0.0%)	0/3 (0.0%)	0/1 (0.0%)	0/1 (0.0%)	1/2 (50.0%)
Oculomotor disturbance	3/28 (10.7%)	1/14 (7.1%)	0/8 (0.0%)	0/2 (0.0%)	0/1 (0.0%)	0/3 (0.0%)	0/1 (0.0%)	0/1 (0.0%)	1/2 (50.0%)
Cases requiring ICU admission (average duration days)	7/28 (25.0%) (74)	5/14 (35.7%) (11.2)	1/8 (12.5%) (5)	0/2 (0.0%)	0/1 (0.0%)	0/3 (0.0%)	1/1 (100.0%) (N/A)	1/1 (100.0%) (21)	0/2 (0.0%)

Autoimmune encephalitis patients were at risk of requiring intensive care unit (ICU) admission. ICU admission was required in 25% of anti-NMDA-receptor encephalitis cases with an average duration of stay of 74 days. Other autoimmune encephalitis subgroups also required ICU admission including anti-glycine-receptor (35.7% ICU admissions with average stay 11.2 days) and anti-LGi1encephalitis (12.5% ICU admissions with average stay 5 days). The cases of anti-IgLON5 encephalitis and anti-GFAP encephalitis both required ICU admission.

Investigation results are summarized in Table 3. Aside from the anti-glycine-receptor related disorders more than 96% of cases in the cohort series had antibody testing performed in both serum and CSF. There were 98.3% of cases with a positive antibody in serum. Many of the autoimmune encephalitis subgroups (anti-DPPX, anti-GABA_B_R anti-IgLON5, anti-GFAP, and anti-SOX1) had an antibody present in both serum and CSF in 100% of cases. The anti-NMDA-receptor antibody encephalitis group had one case with antibody positive in CSF but not serum and three cases where the antibody was positive in serum but not CSF. However, two of these three cases had delayed CSF collection of more than 6 weeks after serum collection. Additionally, both cases were administered immunotherapy after the positive serum anti-NMDA-receptor antibody result but before CSF was collected.

**Table 3:** Summary investigation results of autoimmune encephalitis cases by antigenic target.

	**NMDA-R**	**Glycine-R**	**LGi1**	**Caspr2**	**DPPX**	**GABA_**B**_R**	**IgLON5**	**GFAP**	**SOX1**
Antibody present in serum	27/28 (96.4%)	14/14 (100.0%)	8/8 (100.0%)	2/2 (100.0%)	1/1 (100.0%)	3/3 (100.0%)	1/1 (100.0%)	1/1 (100.0%)	2/2 (100.0%)
Antibody present in CSF	25/28 (89.3%)	1/3 (only 3 CSF samples tested for Ab)	5/8 (62.5%)	1/2 (50.0%)	1/1 (100.0%)	3/3 (100.0%)	1/1 (100.0%)	1/1 (100.0%)	2/2 (100.0%)
Cases with CSF elevated protein (range mg/L)	8/28 (28.6%) (200–1,220)	1/14 (7.1%) (200–500)	2/8 (25.0%) (230–700)	1/2 (50.0%) (270–2,600)	0/1 (0.0%) (350)	0/3 (0.0%) (190–400)	1/1 (100.0%) (850)	1/1 (100.0%) (1000)	2/2 (100.0%) (640–2,400)
Cases with CSF elevated WCC (range 106/L)	8/28 (28.6%) (<1–840)	2/14 (14.3%) (<1–44)	1/8 (12.5%) (<1–16)	1/2 (50.0%) (<1–2,600)	1/1 (100.0%) (15)	3/3 (100.0%) (6–16)	0/1 (0.0%) (<1)	0/1 (0.0%) (1)	1/2 (50.0%) (1–114)
Cases with CSF unavailable	1/28 (3.6%)	5/14 (35.7%)	0/8 (0.0%)	0/2 (0.0%)	0/1 (0.0%)	0/3 (0.0%)	0/1 (0.0%)	0/1 (0.0%)	0/2 (0.0%)
Cases with abnormal brain MRI findings	5/28 (17.9%)	6/14 (42.9%)	4/8 (50.0%)	0/2 (0.0%)	0/1 (0.0%)	1/3 (33.3%)	0/1 (0.0%)	0/1 (0.0%)	2/2 (100.0%)
Cases with a FDG-PET scan performed of the brain	4/28 (14.3%)	0/14 (0.0%)	0/8 (0.0%)	1/2 (50.0%)	0/1 (0.0%)	1/3 (33.3%)	0/1 (0.0%)	0/1 (0.0%)	0/2 (0.0%)
Cases with an abnormal FDG-PET scan of the brain	1/28 (3.6%)	0/14 (0.0%)	0/8 (0.0%)	0/2 (0.0%)	0/1 (0.0%)	0/3 (0.0%)	0/1 (0.0%)	0/1 (0.0%)	0/2 (0.0%)
Cases with abnormal EEG	18/28 (64.3%)	8/14 (57.1%)	4/8 (50.0%)	2/2 (100.0%)	0/1 (0.0%)	2/3 (66.7%)	0/1 (0.0%)	1/1 (100.0%)	1/2 (50.0%)
Cases with seizure recorded on EEG	1/28 (3.6%)	4/14 (28.6%)	1/8 (12.5%)	0/2 (0.0%)	0/1 (0.0%)	0/3 (0.0%)	0/1 (0.0%)	0/1 (0.0%)	0/2 (0.0%)
Cases with epileptiform changes on EEG	4/28 (14.3%)	6/14 (42.9%)	2/8 (25.0%)	0/2 (0.0%)	0/1 (0.0%)	1/3 (33.3%)	0/1 (0.0%)	0/1 (0.0%)	1/2 (50.0%)

The Anti-NMDA-receptor encephalitis group had abnormal magnetic resonance imaging (MRI) in 17.9% of cases. There were only four total cases with MRI findings of increased T2/FLAIR signal or contrast enhancement with most of these abnormal findings in the temporal or frontal lobes. The rate of abnormal MRI scans was higher in other subgroups including anti-glycine, anti-LGi1, anti-Caspr2, and anti-SOX1. Positron emission tomography (PET) was performed in 14.3% of anti-NMDA-receptor encephalitis cases with only one abnormal result which was relatively reduced metabolism within almost the whole brain. PET scans performed on individual cases of anti-Caspr2 antibody encephalitis and anti-GABA-B antibody encephalitis were reported as within normal limits.

Electroencephalogram (EEG) results were abnormal in 50% or more of anti-NMDA-receptor, anti-glycine, anti-LGi1, anti-Caspr2, anti-GABA_B_R, anti-GFAP, and anti-SOX1 cases. These results most commonly represented generalized or focal slowing. Table 3 shows the percentage of cases with a recorded seizure or epileptiform activity on the EEG. Epileptiform discharges (42.9%) and EEG recorded seizure/s (28.6%) were most common in the anti-glycine-receptor antibody group.

**Figure 4:**
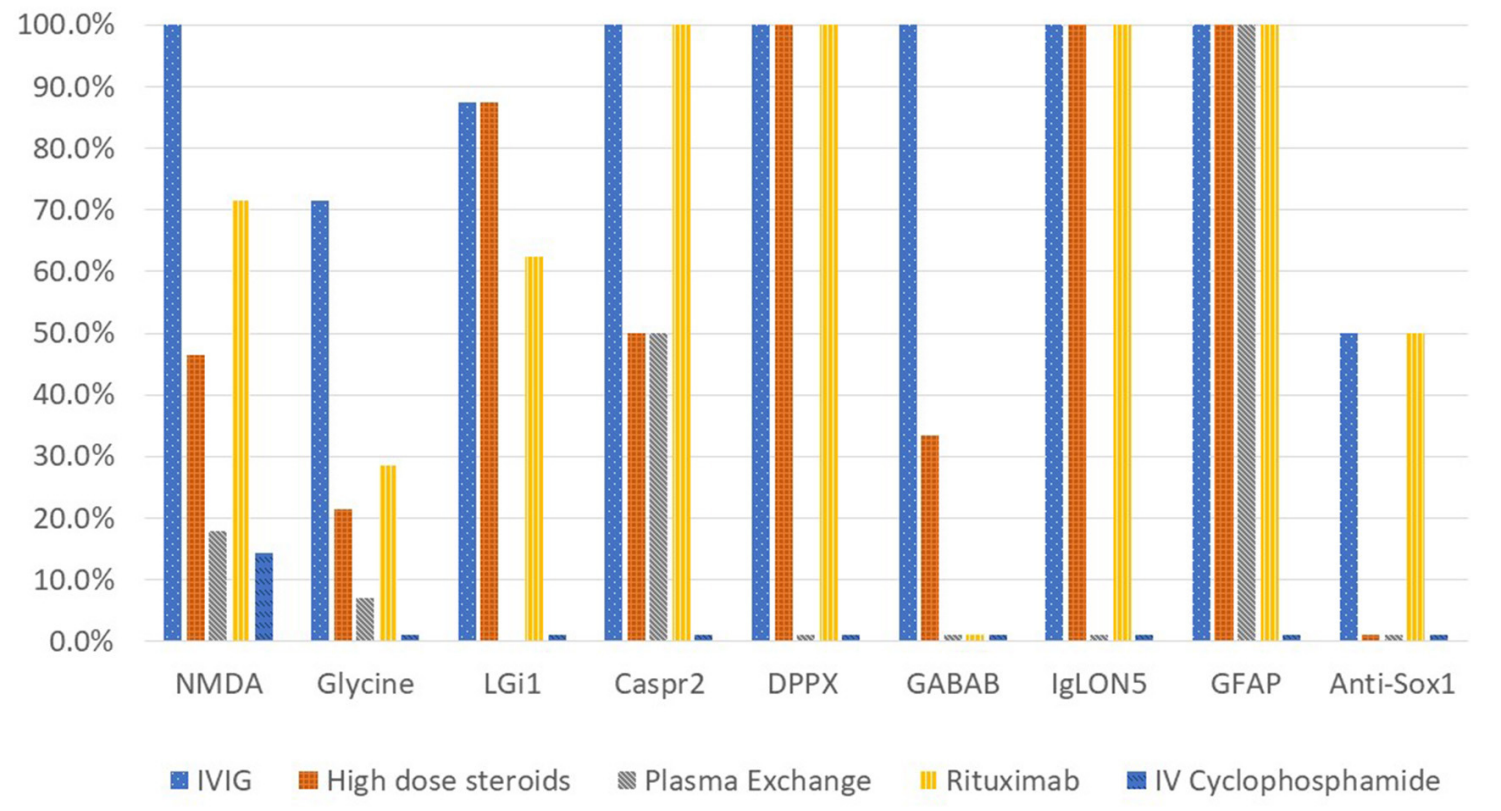
Percentage of autoimmune encephalitis cases receiving immunotherapy.

Immune therapy was administered in fifty-six cases (90.3%) of autoimmune encephalitis in this cohort. Figure 4 shows the different immunotherapies utilized in each autoimmune encephalitis subgroup. Intravenous immunoglobulin (IVIG), high dose steroid therapy (intravenous methylprednisolone), and rituximab were the most utilized therapies. All anti-NMDA-receptor encephalitis cases received IVIG, high dose steroids or plasma exchange as first line therapy, with sixteen cases receiving a combination of these agents. There were 71.4% of anti-NMDA-receptor encephalitis patients who received rituximab and 14.3% who received intravenous pulse cyclophosphamide. The anti-Caspr2, anti-DPPX, anti-IgLON5, and anti-GFAP cases all received both IVIG and rituximab. Resection of an identified ovarian teratoma occurred in 17.9% of cases with anti-NMDA-receptor encephalitis and in one case with a positive anti-glycine-receptor antibody. Additionally, four cases in the anti-glycine-receptor antibody subgroup did not receive immune therapy due to management with other treatments such as anti-epileptics. All patients with anti-GABA_B_R encephalitis received chemotherapy for their underlying small cell lung cancer.

There was documented response to immune therapy in 71.4% of cases in the anti-NMDA-receptor encephalitis subgroup, 71.4% in the anti-glycine-receptor subgroup and 87.5% in the anti-LGi1 subgroup. One of the SOX1 cases and each of the anti-Caspr2, anti-DPPX, anti-IgLON5, anti-GFAP, and GABA_B_R cases responded to immune therapy.

Autoimmune encephalitis cases within the cohort study experienced significant morbidity often with prolonged admissions. The mean number of days till discharge in the anti-NMDA-receptor encephalitis group was 73.96 days. Mean and median times to discharge across the cohort are given in Table 4.

**Table 4:** Summary of treatment and outcome results of autoimmune encephalitis cases by antigenic target.

	**NMDA-R**	**Glycine-R**	**LGi1**	**Caspr2**	**DPPX**	**GABA_**B**_R**	**IgLON5**	**GFAP**	**SOX1**
Average time to discharge (mean days)	74	20.78	36.5	112.5	233	16	165	74	6
Average time to discharge (median days)	28	9	23	112.5	233	17	165	74	6
Cases with full recovery	5/28 (17.9%)	6/14 (42.9%)	3/8 (37.5%)	0/2 (0.0%)	0/1 (0.0%)	0/3 (0.0%)	0/1 (0.0%)	0/1 (0.0%)	1/2 (50.0%)
Cases with partial recovery	22/28 (78.6%)	3/14 (21.4%)	5/8 (62.5%)	2/2 (100.0%)	1/1 (100.0%)	3/3 (100.0%)	1/1 (100.0%)	1/1 (100.0%)	1/2 (50.0%)
Cases with no recovery	1/28 (3.6%)	5/14 (35.7%)	0/8 (0.0%)	0/2 (0.0%)	0/1 (0.0%)	0/3 (0.0%)	0/1 (0.0%)	0/1 (0.0%)	0/2 (0.0%)
Deaths	0/30 (0.0%)	1/14 (7.1%)	0/8 (0.0%)	0/2 (0.0%)	0/1 (0.0%)	0/3 (0.0%)	0/1 (0.0%)	0/1 (0.0%)	0/2 (0.0%)
Average baseline mRS score	0.19	0.21	0	0	0	0.33	1	0	0
Average maximum mRS score	3.78	6	3.65	5	5	3	5	5	2.5
Average final mRS score (at last follow-up)	1.07	1.28	0.75	2	1	1.33	3	2	1

The modified rankin scale (mRS) (29) was used to assess premorbid status, status at the most severely impaired stage of the disease course and the status at last follow-up. The average follow-up was >12 months across the cohort. The average mRS showed that there was little disability at baseline across all subgroups. At maximal impairment the anti-NMDA-receptor cohort group had an average mRS of 3.78 representing moderate to moderately-severe disability. The anti-LGi1 and anti-glycine subgroups were similar to the anti-NMDA-receptor group. The anti-SOX1 cases showed the lowest average disability at maximum disease severity (mRS 2.5). The anti-Caspr2, anti-DPPX, anti-IgLON5, and anti-GFAP cases were on average more severely affected at maximal disease severity when compared to the anti-NMDA-receptor group.

Most patients in the cohort improved but continued to experience symptoms. The average final mRS in the anti-NMDA-receptor encephalitis group was 1.07 with a final mRS range across the cohort of 0.75 to 3. There was one death recorded, in a patient with anti-glycine-receptor antibody related disease (PERM), due to disease progression.

## Discussion

This cohort study adds to the growing body of literature describing autoimmune encephalitis, detailing a state-based Australian experience. It establishes that there are a significant number of cases (sixty) of autoimmune encephalitis diagnosed and managed through Queensland public hospitals, of which Anti-NMDA-receptor encephalitis was the most common subtype. Represented in smaller but still significant numbers were other subgroups of autoimmune encephalitis with other antigenic targets including glycine receptor, LGi1, Caspr2, DPPX, GABA-B, IgLON5, GFAP, and SOX1. Also illustrated in this cohort is the rapid growth in the number of cases diagnosed, most prominently seen in the anti-NMDA-receptor encephalitis group in the period 2017 to 2019. This growth is likely representative of improved awareness of autoimmune encephalitis, increased local availability of anti-NMDA-receptor antibody testing, and the evolution and more widespread usage of screening guidelines for mental health and epilepsy presentations such as the recommendation for autoimmune encephalitis screening in selected psychosis cases in Queensland since 2016.

When first described, anti-NMDA-receptor encephalitis was predominantly a disorder of young females with a high percentage of cases with an ovarian teratoma (1, 20, 30). The female predominance, younger age of onset, and association with teratoma is present in this cohort. However, the overall cohort of anti-NMDA-receptor encephalitis also demonstrates a growing male representation (45%) and that 90% of cases did not have an associated teratoma or other malignancy.

The wide range and heterogeneous nature of the symptoms displayed at presentation and across the disease course demonstrates that the Queensland autoimmune encephalitis cohort comprises a complex and medically challenging group. Investigations such as MRI, EEG, and less commonly PET scans are largely either normal or non-specific and illustrate the lack of a defining biomarker as referenced in the 2016 consensus diagnostic criteria (31).

Analyzing cases of autoimmune encephalitis from across an entire state/service district makes this dataset distinct from recent studies analyzing experience at a single center (21, 22), and prior larger studies where cases were recruited through referrals from multiple health districts to a reference laboratory (22). This regional approach included cases that may come from non-tertiary centers, psychiatry and oncology services. When compared to recent single center studies, our Queensland dataset shows similar outcome mRS for the anti-NMDA-receptor subgroup (21), a comparable utilization of multiple immune therapies (22), a high proportion of cases with psychiatric symptoms (22) and a similar low mortality when compared with larger referral cohorts (20). Our cohort also correlates with recent evidence regarding the high morbidity and significant health care resources required to manage patients with autoimmune encephalitis (23). This is illustrated in this cohort by prolonged hospital admissions (which often require additional ICU admission), the need for combination immunotherapy and multiple investigations such as MRI, EEG, and PET.

The limitations of this study are the retrospective nature and relatively small number of cases available for analysis. This is inherent when studying a rare disorder in a defined geographic area such as Queensland, Australia. There was one case where atypically the anti-NMDA-receptor antibody was present in serum but not CSF where this was not accounted for by CSF testing delay and treatment prior to CSF antibody testing. This patient presented with psychiatric symptoms and responded to immune therapy. Although atypical, cases presenting with psychiatric symptoms have previously been reported with positive serum ant-NMDA-receptor antibody studies but negative CSF antibody studies (32).

With regard to management and clinical outcomes, combination immunotherapy appears to be effective, but the documented improvement does not correlate with short hospital admissions. This is contributed to by the severe nature of autoimmune encephalitis as evidenced by the maximal mRS scores ranging from 2.5 to 6 and the average score in the anti-NMDA-receptor encephalitis group of 3.78. The clinical improvement displayed across this cohort at last follow-up is encouraging, but demonstrates that patients are often left with ongoing symptoms and in many cases mild disability as evidenced by an average mRS score at last follow-up of >0.75 across all groups.

## Conclusion

In conclusion, autoimmune encephalitis in Queensland is an increasingly common disorder with a rapid recent escalation in the number of diagnosed cases. The diagnosis of these conditions continues to be highly dependent on the availability of specialized autoantibody testing.

The 60 cases of autoimmune encephalitis represented in this study are small when compared to more prevalent neurological disorders such as stroke or migraine. However, these cases are significant as autoimmune encephalitis presented with a complex interplay of heterogeneous symptoms, lacked clear neuroimaging findings and was associated with high morbidity and significant health care system burden.

What is currently not known is whether this increased case detection of autoimmune encephalitis will now plateau, as the majority of cases are now being identified. The alternative is that the growth demonstrated in this cohort study will continue with further advances in understanding of this disorder. These advances may come via more effective implementation of testing criteria, better identification of potential cases or the emergence of groups of patients where autoimmune encephalitis accounts for a percentage of presentations.

## Data Availability Statement

The raw data supporting the conclusions of this article will be made available by the authors, without undue reservation.

## Ethics Statement

The studies involving human participants were reviewed and approved by Metro-South Human Research and Ethics Committee reference HREC/17/QPAH/423. Written informed consent for participation was not required for this study in accordance with the national legislation and the institutional requirements.

## Author Contributions

AS and SBl designed the study. AS and NW performed the data collection. AS performed the analysis and wrote the paper. KP, DG and RW contributed to the methodology with regard to laboratory based testing as well as reviewing and editing the manuscript. CO'G, BT, CM, SBl, RA, PM, and SBl provided additional data and reviewed and edited the manuscript. All authors read and approved the manuscript.

## Conflict of Interest

The authors declare that the research was conducted in the absence of any commercial or financial relationships that could be construed as a potential conflict of interest.
